# Chromosomal scale assembly reveals localized structural variants in avian caecal coccidian parasite *Eimeria tenella*

**DOI:** 10.1038/s41598-023-50117-0

**Published:** 2023-12-20

**Authors:** Subodh K. Srivastava, Carolyn Parker, Celia N. O’Brien, Matthew S. Tucker, Peter C. Thompson, Benjamin M. Rosenthal, Jitender P. Dubey, Asis Khan, Mark C. Jenkins

**Affiliations:** USDA-ARS Animal Parasitic Diseases Laboratory, Beltsville Agricultural Research Center, BARC-East Building 1040, 10300 Baltimore Ave., Beltsville, MD 20705 USA

**Keywords:** Computational biology and bioinformatics, Genetics, Molecular biology, Zoology

## Abstract

*Eimeria tenella* is a major cause of caecal coccidiosis in commercial poultry chickens worldwide. Here, we report chromosomal scale assembly of *Eimeria tenella* strain APU2, a strain isolated from commercial broiler chickens in the U.S. We obtained 100× sequencing Oxford Nanopore Technology (ONT) and more than 800× Coverage of Illumina Next-Seq. We created the assembly using the hybrid approach implemented in MaSuRCA, achieving a contiguous 51.34 Mb chromosomal-scale scaffolding enabling identification of structural variations. The AUGUSTUS pipeline predicted 8060 genes, and BUSCO deemed the genomes 99% complete; 6278 (78%) genes were annotated with Pfam domains, and 1395 genes were assigned GO-terms. Comparing *E. tenella* strains (APU2, US isolate and Houghton, UK isolate) derived Houghton strain of *E. tenella* revealed 62,905 high stringency differences, of which 45,322 are single nucleotide polymorphisms (SNPs) (0.088%). The rate of transitions/transversions among the SNPs are 1.63 ts/tv. The strains possess conserved gene order but have profound sequence heterogeneity in a several chromosomal segments (chr 2, 11 and 15). Genic and intergenic variation in defined gene families was evaluated between the two strains to possibly identify sequences under selection. The average genic nucleotide diversity of 2.8 with average 2 kb gene length (0.145%) at genic level. We examined population structure using available *E. tenella* sequences in NCBI, revealing that the two *E. tenella* isolates from the U.S. (*E. tenella* APU2 and Wisconsin, “ERR296879”) share a common maternal inheritance with the *E. tenella* Houghton. Our chromosomal level assembly promotes insight into *Eimeria* biology and evolution, hastening drug discovery and vaccine development.

## Introduction

*Eimeria* species, the causative agent for avian coccidiosis, are protozoa parasitizing a wide array of vertebrate and invertebrate hosts, including livestock. Avian coccidiosis, widespread in poultry, causes more than $13 billion in economic damage each year to poultry industries worldwide^1^. For chickens, infection can limit growth, feed conversion, and egg production; severe cases can be fatal. Although there are 7 well known species that infect chickens, *E. tenella* is one of the most pathogenic causing hemorrhagic caecal coccidiosis in chicks. Although, prevention of disease relies on chemoprophylaxis or vaccination with low doses of *Eimeria* oocysts, a recombinant vaccine is an ideal alternative control approach. Several vaccine candidates have been identified (e.g. immune-mapped protein 1 (IMP1), apical membrane antigen 1 (AMA1)) However, population genetic structure of closely related apicomplexan parasites like *Plasmodium* and *Toxoplasma* revealed an extensive diversity in antigen-presenting genes. Hence, understanding the population genetic structure and evolution of *Eimeria* species based on whole genome comparative analysis are critical to developing cost-effective vaccine candidates.

Long-read sequencing technologies can improve genome assembly quality by resolving complex repeats and structural variations^2^. Better assemblies improve the assessment of phenotypic differences derived from structural alteration. However, whole genome sequencing of *Eimeria* genomes lags behind many other closely related apicomplexan parasites due to its tough oocyst wall leading to low quality and quantity genomic DNA which is poorly suited to long-read sequencing^3^. Previous assemblies were limited to sanger sequencing and second-generation sequencing and 454 sequencing (Roche Applied Science), which made de novo assembly difficult. The first-generation assemblies of *E. tenella* (Houghton) were fragmented into thousands of contigs and subsequent assemblies of other species using Illumina short-read technology were little better^4,5^. Recently, the complete genome sequence of *E. tenella* (Houghton) was constructed with a 41-fold coverage from Pacific Biosciences long reads and 107-fold coverage from 10× Genomics reads of *E. tenella* Houghton parasites. This assembly consists of 15 chromosomal pseudomolecules, spanning 53.25 Mb^6^. The genome, although of great use, developing strategies to combat avian coccidiosis requires high-quality genomic resources and an understanding of the degree and nature of variation between isolates and geographic locations. Hence, in the current study, we present chromosomal level *E. tenella* APU2 genome sequence of an isolate using long-read Oxford Nanopore and short-read Illumina sequences. Intraspecific genetic variation shapes interactions among species and helps to understand their arrangement in the biological communities.

Parasites genomes can vary within and among species and these variation leads to functional and non-functional attributes that could either use for a potential marker to mark of identity species for diagnostics purposes or affect organism’s phenotypes and traits. Of sequence variation in coding region can leads to functional loss or gain of genes function that could potentially utilize for improvement^7^. Developing strategies to combat avian coccidiosis thus requires an understanding of the degree and nature of variation between isolates and geographic locations, both in the primary genome sequence (SNPs and indels) and in larger structural variants. Here, we present chromosomal scale *E. tenella* genome sequence of an isolate (APU2) from commercial broiler chickens in the U.S. using long-read Oxford Nanopore and short-read Illumina sequences. By comparing two strains (*E. tenella* Houghton and *E tenella* APU2) for gene content and structural rearrangements, we sought to elucidate the regions of the *E. tenella* chromosome may be under selective pressure. Additionally, we employed comparative genomics using other publicly available *Eimeria* sequences to define localized structural variations and show how occasional, consequential recombination shapes the population genetic structure of *E. tenella*.

## Results and discussion

### Genome assembly and chromosomal scaffolds

**Figure 1:**
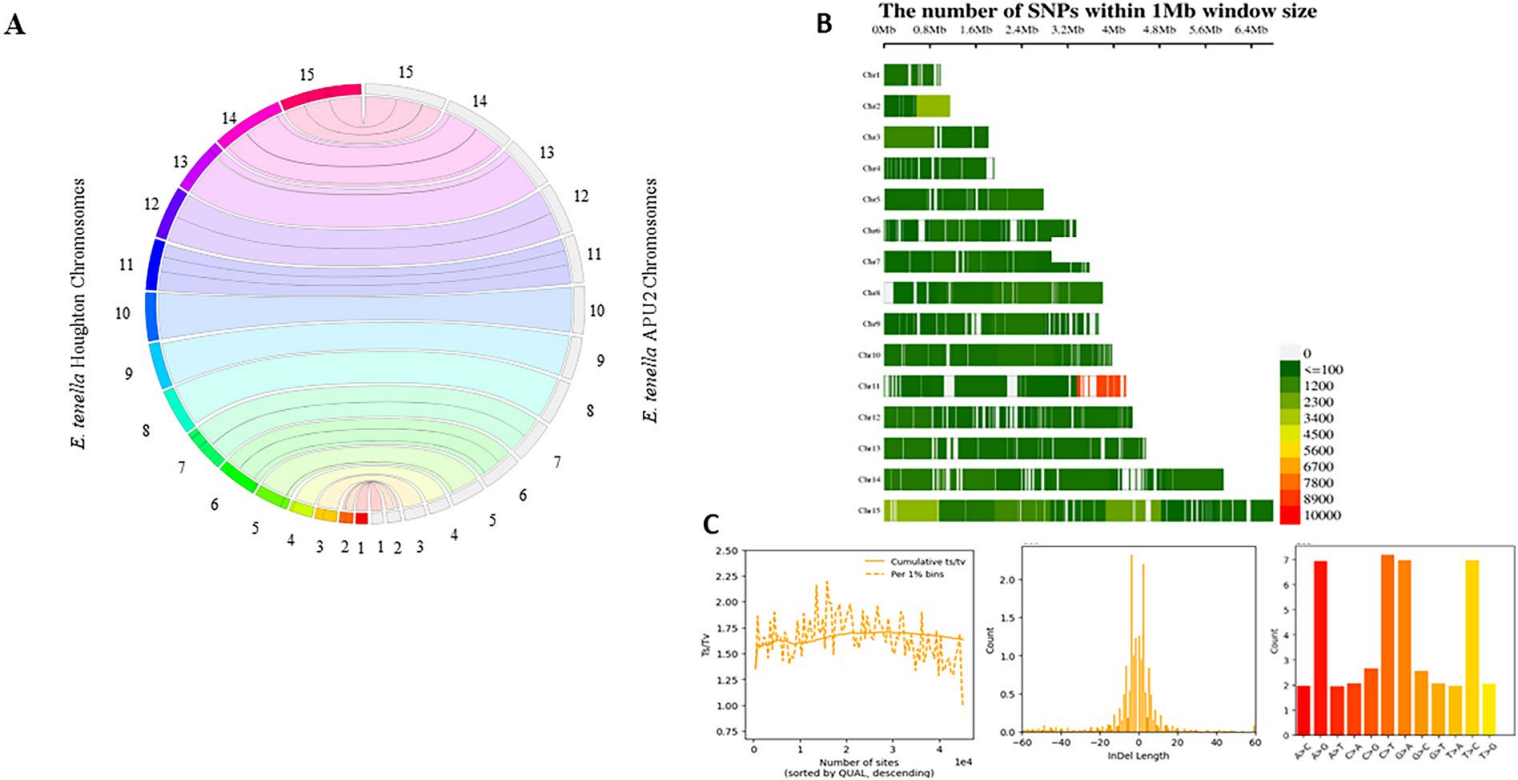
(**A**) Chromosomal consistency plot comparing *E. tenella* Houghton (as reference) and *E. tenella* APU2 (sequenced). (**B**) Single Nucleotide Polymorphisms (SNPs) on *E. tenella* Houghton chromosomes with NGS of *E. tenella* APU2. Each bin size with colored dark to light green and red represents the number of SNPs in 1 Mb window size according to density of SNPs. Fewer than 100 SNPs/Mb occurred in most regions (depicted as dark green). SNP density generally appeared to vary randomly from the mean when plotted at 1000 bp increments across the chromosomes. (**C**) Plotted SNPs with cumulative ts/tv, Indel distribution and Nucleotide substitution across the chromosomes with 1^e−3^ scale.

We assembled the *E. tenella* APU-2 genome using an adaptive strategy incorporating evidence from both short and long reads, thereby reducing the number of contigs and errors^3,8^. A summary of the assembly statistics can be found in Table 1 and a graphical representation of the workflow is provided in the supplemental information (Fig. S1). We generated 975,352 ONT long reads which averaged 6 kb and an average read quality of 12, yielding an N50 initial read length of 10,228 bp. The 88% of reads passed our quality filter of > 7 Q-score and were converted to fastq format for further processing^9,10^. We processed 856K quality filtered long reads derived from Oxford Nanopore Technology (ONT) and 307 million reads (PE) derived from Illumina Next Seq technology; combined evidence from more than 100× (ONT) and 800× (Next-Seq) coverage achieved with the sequencing approach (Table 1). The MaSuRCA assembler produced a total of 187 contigs, just 6 of which encompassed more than 50% of the data (L50); the contigs containing half of the assembly averaged almost 4 million bases (N50 = 3,921,563)^11^. The assembly and analyses workflow with genome completeness statistics are listed in Table 1 (Fig. S1). The reference assembly from the Houghton strain supported further contig orientation and scaffolding^6,11^, enabling us to achieve 15 chromosomal scaffolds incorporating 51,348,175 bases. This chromosomal assembly contained just 114 gaps that aligned well with the Houghton genome (Fig. 1A). Employing BUSCO with coccidian dataset (coccidia_odb10)^9,12^, identified 496 of 502 expected single copy orthologs as complete (Fig. S2).

**Table 1:** Assembly statistics of *E. tenella* APU2 compared with *E. tenella* Houghton strain.

Attributes	*E. tenella* APU2
Assembly name	APDL-v1.0
Total ONT reads	975,353
Total NextSeq reads	307,760,588
Quality total reads	856,272
Cumulative coverage	107×
Read mean length	6117
Chromosome	15
L50	6
Shortest sequence	989,717
N50	3,922,363
Longest sequence	6,756,684
Sum	51,306,473
Number of gaps	114
GC%	51.71
Assemble completeness (CEGMA)	80%
Assemble completeness (BUSCO)	99%
Annotated genes	8060
NCBI Acc	CP118642-CP118656

### Gene prediction and functional annotation

We used various annotation pipelines on ordered assemble chromosomal-scale scaffolds, and supported gene prediction using AUGUSTUS with RNA-seq support^10,13^. Predicted genes were annotated according to functional domains using the Pfam database^14,15^. A further scan the genome for tRNA using the tRNAscan-SE annotation pipeline identified 326 tRNA encoded in the *E. tenella* APU2 genome^16^. We examined alternative splicing of genes in the APU2 using AUGUSTUS which uses a Generalized Hidden Markov Model (GHMM) for gene structure^17^. Using RNA-seq data derived from sporulated oocysts of the APU2 strain, we identified predicted 8060 genes, 63,166 exons, 86,880 introns, 9160 transcriptions start/stop sites. Among these 8060 genes derived from primary transcripts, 865 have two alternatives, 173 have three, 49 have four, 11 have five, 3 have six alternative spliced transcripts forms. Those exhibiting alternative forms include genes known, in other Apicomplexa, to undergo regulation via alternative splicing^18^. These include SERRATE_ars2 (PF05540), and RNA polymerase II (involved in transcription of snRNA genes), shows high number of alternative forms^19^. These annotated genes were categorized into biological functional groups and updated with GO-terms^20^. We predicted 8060 genes genome-wide and classified 6278 (78%) genes with Pfam annotations; 1356 were assigned functional GO-terms (Table S2). The predicted genes were classified by functional gene ontology (GO) terms covering various domains of molecular and cellular biology and protein function^21^.

The phylogenetic relationships between gene sequences are necessary to provides the back ground for understanding the evolution and diversity between organisms^22^. The evolutionary insights gained from the chromosomal-level assembly using phylogenic analyses reveal *Eimeria tenella* APU2, *E. tenella* Houghton and *E. necatrix* in same clade (Fig. S6). Orthologous genes investigation between *E. tenella* APU2 with respect to *Toxoplasma gondii* (*T. gondii*) found 4548 orthogroups. When orthogroups analysis was extended to other important avian parasites (*E. mitis*, *E. brunetti*, *E. praecox*, *E. maxima*, *E. acervulina*, *E. tenella* APU2, *E. tenella* Houghton, *E. necatrix*) and other close relatives, mouse coccidium *E. falciformis* and *T. gondii,* that has wide host range, 7658 orthogroups were discovered indicating that all avian Eimeria species are more closely related to each other than to *T. gondii*. Phylogeny suggests a close evolutionary relationship between *E. necatrix* and *E. tenella,* which differs in the severity of the disease they cause as well as the site of their replication. Comparing genomes may ultimately reveal the genetic basis of differences among parasitic species infecting distinct replication sites in the gastrointestinal tract of chickens, and among parasitic species infecting different host species. While dissecting genetic basis of such phenotypic difference lies beyond the scope of this report, we hope that a durable genome assemblies and transcriptomic data will translate to such functional insights^23,24^. The *E. necatrix* has been reported and recognized as the most pathogenic Eimeria species which infects chickens, but *E. tenella* is more common and exerts a greater impact on poultry production^24,25^.

### Localized structural variation.

We identified 45,322 SNPs (0.088% of the genome) with a slight preponderance of transitions over transversions (ts/tv 1.63). In addition, we identified strong evidence for 17,583 indels. With some exceptions, SNPs were evenly distributed across the chromosome. Fewer than 100 SNPs/Mb occurred in most regions (depicted as dark green in Fig. 1B). SNP density generally appeared to vary randomly from the mean when plotted at 1000 bp increments across the chromosomes (Fig. 1B). In stark contrast, we observed regions of chromosomes exhibiting markedly more differences between the two strains. Over 2000 SNPs/Mb occurred in a portion of chromosome 2. Approximately 3000 SNPs/Mb in a portion of chromosome 15, and up to 10,000 in a portion of chromosome 11. These observation support that some degree of structural variation between Houghton and APU2 were localized on few chromosomes. Regions characterized by fewer than 100 SNPs/Mb predominate (and are depicted in green in Fig. 1B).

The predicted *E. tenella* APU2 genes analyzed with ProtVirDB (Protozoan Virulent protein database)^26^. The information provides virulent proteins in different parasitic protozoans and organize them under a unifying classification representation with functional categories^26^. It has been reported that most proteins associated with virulent are either mono- or hetero-repeats (or both) restating the importance of repeats in parasite virulence mechanisms. The analyzed *E. tenella* APU2 predicted genes possess 286 genes that match the virulent proteins includes 7811 SNPs as compared to *E. tenella* Houghton strain. Out of these, 4093 in the intergenic and 3718 were in exonic region could play significant role in protein frame may leads alter pathogenicity in the organisms. The protein-coding genes of transposons domains group were analyzed from Pfam annotation, reveals 16 reverse transcriptase (RNA-dependent DNA polymerase, 48 protease, 13 integrases, 15 RNaseH, 8 gag, and 19 chromodomain proteins incorporate SNPs between APU2 and Houghton strain presented in Table S2.

One of the sequence features is dinucleotide pattern C followed by G (CpG sites) tend to occur less frequently than what would be expected given the frequency of those bases in a genome. To investigate whether the two strains of *E. tenella* share common distributions of CpG, we identified CpG islands (defined as regions, at least 200 bp long, with a C + G content of 50% (or more) in the stretch of DNA and, an CpG observed/expected, in excess of 0.6 was analyzed between *E. tenella* Houghton reference and *E. tenella* APU2 to compared genome-wide^27^. Nearly equivalent numbers were identified 28,527 CpG-sites in *E. tenella* Houghton as compared to 27,533 CpG-sites in *E. tenella* APU2 and the chromosomal distribution appeared comparable (Fig. S4).

**Figure 2:**
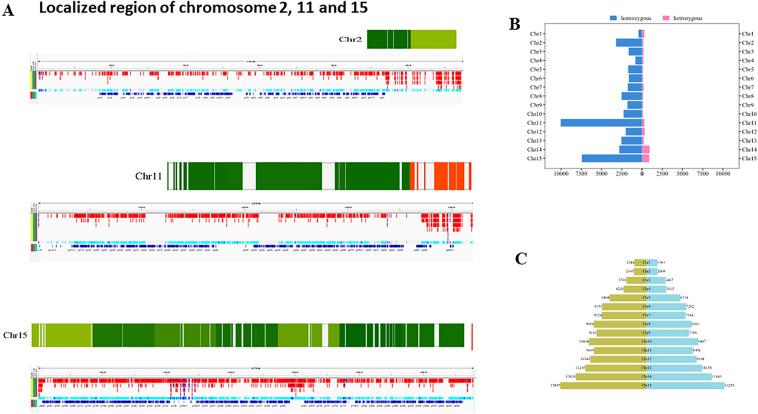
(**A**) Visualization of selected chromosome 2, chromosome 11 and chromosome 15 with patchy hypervariable regions and density of predicted genes cross the regions showing localized variations. (**B**) Analysis of heterozygous and homozygous class of SNPs across the chromosomes. (**C**) Chromosome wise repeat number in both the isolates (*E. tenella* Houghton and *E. tenella* APU2). The identified 125,252 and 11,350 repetitive regions in the Houghton and APU-2 strains of *E. tenella* by using Red (REpeat Detector) across chromosomes.

We sequenced populations of haploid parasites and used SNP detection tools GATK, employing stringent parameter thresholds, that take into consideration both the frequency of each allele and the quality of each basecall to limit the False Discovery Rate otherwise introduced by sequencing errors. “Heterozygous” positions constitute those positions for which strong evidence exists for the existence of more than one allele in the sequenced population. For statistical consistency, we analyzed only those positions reliably determined to bi-allelic SNPs. We classified each SNP and indel as either homozygous or heterozygous and as genic or intergenic (Fig. 2B). We identified a total of 62,905 strongly supported differences (45,322 SNPs + 17,583 indels). More (39,471) occurred in 35.09 Mb intergenic regions than in 16.25 Mb genic (exonic and intronic) regions (23,028) (meaning the rate of differences in remaining genomic regions was 1124 differences per Mb and 1417 per Mb in the intergenic and genic regions, respectively). These reveal that out of 8060 predicted genes, at least 5949 genes possess at least one SNP or Indel.

Genome variation in eukaryotic pathogens underpins both fundamental biology, such as the ability of the parasite to evade the human immune response, and clinical outcomes, through the evolution of drug resistance^28^. Repetitive regions can shape genome evolution in various ways^28^. The Eimeria species revealed alternating regions of repeat-poor and repeat-rich sequences in all chromosomes of *E. tenella* and across the genomes of all *Eimeria* species^5,28^. We identified 125,252 and 111,350 repetitive regions in the Houghton and APU-2 strains of *E. tenella ,* depicting their distribution using Red (REpeat Detector)^29^ (Fig. 2C). In spite of equal distribution according to chromosomes, more repeats occurred in few chromosomes of the Houghton strain than in the APU2 strain. We investigated the distribution of simple sequence repeats (SSRs) longer than 12nt in the *E. tenella* APU2 genome using the PERF prediction tool^30^, identifying 152,629 repetitive trimers, 24,407 tetramers, 12,886 pentamers and 43,777 hexamers. These repetitive sequences expand and contract dynamically and are generally among the most rapidly changing sequences in the genome. These repeats have long been used for a variety of purposes in the areas of population genetics, and marker-assisted selection.

We further assessed these differences in relation to Pfam and GO-teams to explore their biological function. Regions harboring excessive variation (chromosome 2, 11 and 15) harbor comparatively fewer genes, although they contain a few important genes i.e., PI4 Kinases Phosphatidylinositol 3- and 4-kinases, AIM3 Alte, red inheritance protein, Pkinase domains, DDHD domain, Hemagglutinin, and Asp proteases on localized variable region of chromosome 11. We evaluated the number of genes predicted especially at the region showing high nucleotide diversity (hypervariable) towards telomeres. There were no significant genes found at the ends of other genome regions with higher concentrations of SNPs (Fig. 2A).

### Gene families enriched with SNPs variation

We focused on 30 highly variable Pfams (including Pkinases, SUIM assoc, TFIIA, SR-25, RskA, RRM_1, WD40 and surface antigens in the SAG family^31^). 54 and 50 genes with SAG domain-containing proteins occurred in the APU2 and Houghton strains, respectively. These contain SNPs in intergenic and intragenic positions (Table S3). Likewise, we evaluated 30 Pfams with domains differing evidently between the two strains of *E. tenella* (Fig. S3). These genes encompass SNPs that may influence protein function. Table S3 summarizes these SNPs that are distributed among Intergenic, Exonic and CDS.

### Population studies with other *Eimeria tenella* strains

We made use of a broader array of publicly available genome sequences to assess more general patterns of genome variation, accepting a lower threshold of evidence to draw inferences from less-deeply sequenced genomes. Discerning the structure of *Eimeria* populations may be complicated by multi-species co-infections, strain-specific immunity, strain-specific antigenic polymorphism, rapid *Eimeria* cycling, and rapid evolution influenced by varying levels of fecundity and pathogenicity^32^.

The paucity of genetic markers constrain prior population-genetic studies^33^; nonetheless, African, Indian, and Nigerian *E. tenella* strains characterized using a Sequenom MassARRAY SNP panel genotyping, 55 SNPs identified considerable genetic diversity and significant linkage disequilibrium (LD). Hence, we compared the genetic diversity of the U.S. strains, particularly our *E. tenella* strain APU2 (ET_S13), and with other publicly available *Eimeria* sequences in the Sequence Read Archive (https://www.ncbi.nlm.nih.gov/sra) using genome-wide short read sequence data to understand the population genetic structure of *E. tenella* (Table S1).

To quantify the genetic diversity among available *E. tenella* genomes, we mapped the nuclear genomes of *E. tenella* to the reference, identifying a total of 76,549 high-quality bi-allelic SNPs sustained by at least 10× coverage (Table S1). For statistical consistency, we analyzed only those positions reliably determined to bi-allelic SNPs in all sequences.

**Figure 3:**
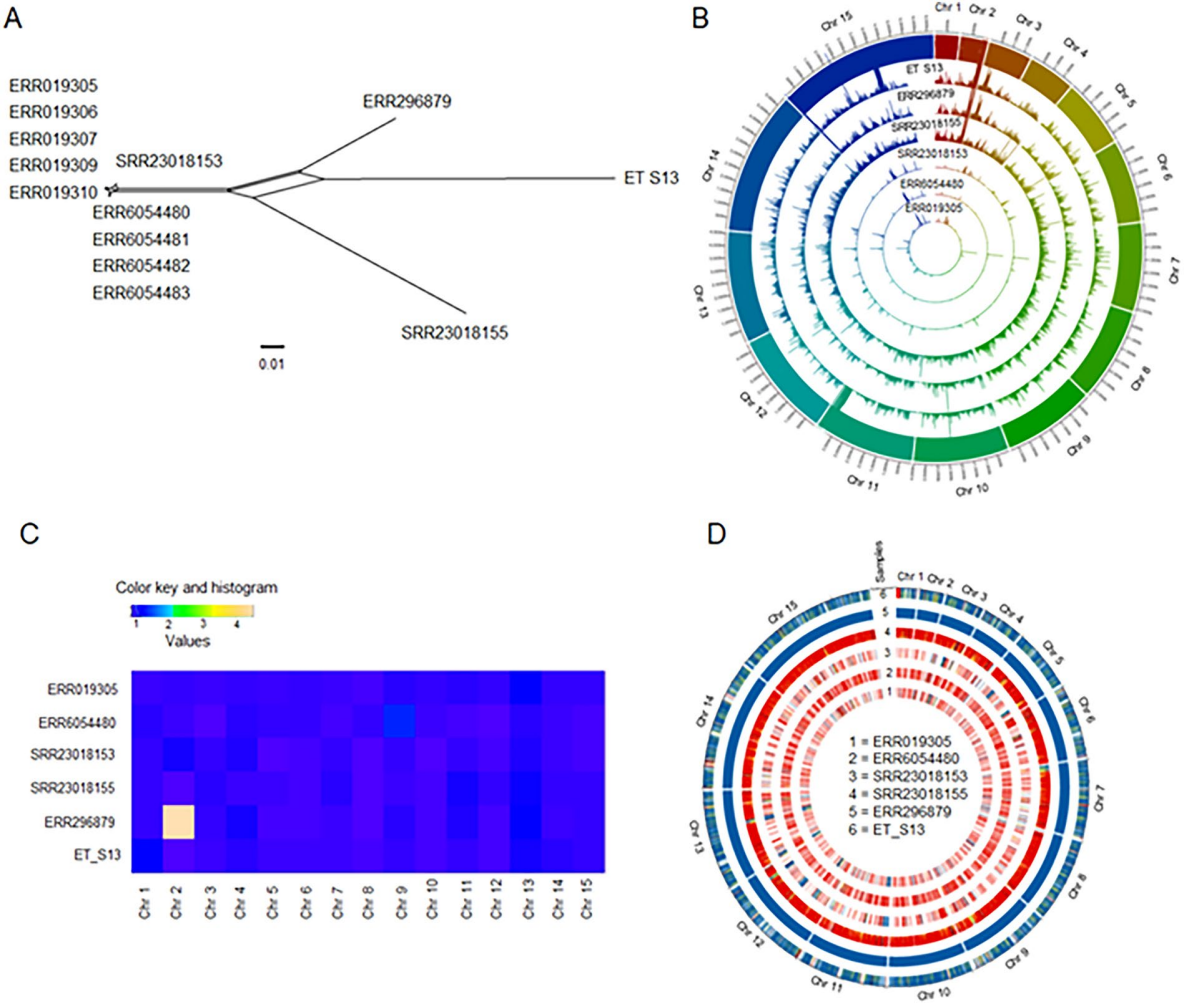
Genetic diversity of nuclear genomes of *E. tenella*. (**A**) Population genetic structure of *E. tenella*. A neighbor-net analysis was performed using genome-wide SNPs (77,648) without gaps and Indels. Houghton strain sequences were clustered tightly within a single node, however, the USA strains (ET_S13 and ERR296879) were distantly related to each other and from Houghton strain sequences. The scale bar indicates the number of SNPs per site. (**B**) Circos plot depicting the genome-wide genetic diversity of *E. tenella* strains. Histograms present in each track show the total number of SNPs present in 5 kb sliding windows. Each track represents each *E. tenella* strain. The chromosome name and the corresponding scaffolding are depicted in the outmost track. SNPs were identified by reference mapping using the reference strain and variant calling using the GATK pipeline^61^. (**C**) Heat map of the ploidies for the *E. tenella* sequences indicates that most chromosomes are near haploid except the chr 2 of ERR296879. (**D**) The Circos-plot of the genome-wide distribution of heterozygous and homozygous SNPs in 5 kb sliding windows. The U.S. strains *E. tenella* APU2 “ET_S13” and Wisconsin, “ERR296879” showed long stretches of homozygous blocks, whereas other strains showed long stretches of heterozygous blocks, indicating the presence of mixed alleles in sequences. Red color = > 90% of heterozygous SNPs, blue = > 90% of homozygous SNPs, yellow = 50% heterozygous, 50% homozygous SNPs. Each track represents a single genome sequence.

A network constructed from these SNPs tightly clustered all Houghton sequences in a single node, indicating that these sequences were derived from the same strain (Fig. 3A). Strikingly, both U.S. isolates were separated not only from Houghton strains but also from each other. Surprisingly, one of the isolates from China but attributed to the Houghton strain (SRR23018155) (Table S1) was determined to be quite distinct from those derived from the United Kingdom and ascribed as Houghton strains (Fig. 3A).

The neighbor network helped visualize gene flow among strains. To establish the extent of polymorphism across chromosomes and strains, we developed a Circos-SNP plot by calculating the total number of SNPs present in 5 kb sliding windows (Fig. 3D). The SNP plot closely resembled the neighbor network analysis, distinguishing the U.S. strains from the Houghton strains. SNPs were distributed uniformly throughout the chromosomes and more densely in sub telomeric regions, indicating segmental duplications occurring in those regions (Fig. 3B).

Interestingly, we identified large haploblocks in the U.S. strains that resemble Houghton strains, interspersed with highly divergent regions. Hence, based on network analysis and SNPs plots (Fig. 3B,D), we conclude that Houghton and the U.S. strains have diverged but retain shared ancestral blocks, perhaps owing to local admixture.

To understand the role of evolutionary pressures that account for the highly localized genetic diversity between the U.S. strains and the Houghton strains, we determined the ploidy (Fig. 3C) and degree of heterozygosity (Fig. 3D) occurring in these strains. *E. tenella* sequences appear haploid, with little to no evidence of aneuploidy except on chromosome 2 of ERR296879.

Although haploid, polyclonal populations could conceivably contribute to polymorphism in such genome sequences. Thus, we calculated the genome-wide heterozygosity (blue-colored blocks) and heterozygosity (red-colored blocks) among the *E. tenella* sequences (Fig. 3D) in 5 kb sliding windows. All the Houghton sequences contained SNP-poor heterozygous blocks, likely derived from sequencing of non-clonal populations. By contrast, the U.S. Wisconsin strain (ERR296879) appeared homozygous genome-wide (with one aneuploid chromosome); the APU2 strain is generally lacking in heterozygosity but does include a few long runs of heterozygous blocks (possibly due to sequencing a non-clonal population) located in sub-telomeric regions; further supporting the segmental evolution of these parts of the genome. Notably, the strain ascribed to the Houghton strain SRR23018155 and sequenced in China, showed long runs of SNP-dense heterozygous blocks throughout the genome. Such long runs of heterozygosity in a haploid organism indicate that this sequence derived from a mixed, genetically variable population of parasites.

**Figure 4:**
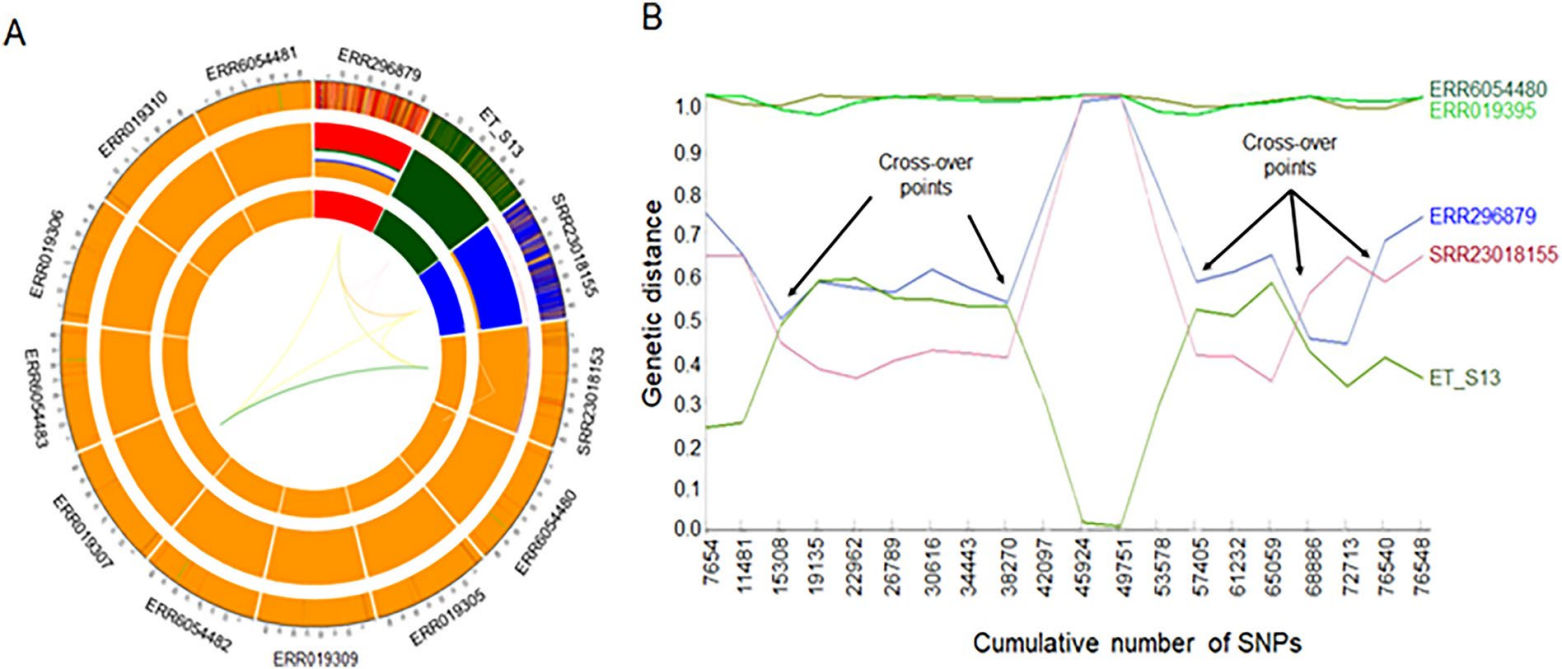
Admixture analysis of the *E. tenella* genome. (**A**) Population genetic structure and admixture clustering of *E. tenella* genomes using POPSICLE with current population number K = 4, represented in the innermost circle of the Circos plot. The middle track indicates the relative percentage of shared ancestry within each genome, whereas the outmost track represents the genome-wide admixture profile of sequences in 5 kb blocks. The thickness of the connecting lines varies with the percentage of shared ancestry. (**B**) Recombination analysis based on incongruence in genetic distance after pairwise comparison of SNPs present across the *E. tenella* genomes. Arrows indicate the cross-over points. The line on the graph demonstrates the genetic distance of each sequence (y-axis), whereas the x-axis represents the total number of SNPs.

To better depict the shared ancestry pattern among the Houghton and the U.S. strains, we reconstructed population genetic structure using POPSICLE^34^. We estimated the number of supported ancestries (K) as 4 using the Dunn index^35^. The POPSICLE plot represented each clade (the inner circle) by a unique color; the plot also depicts haploblocks (the middle circle), and detailed chromosome painting, in 5 kb sliding windows of shared ancestry, to reveal the pattern of local admixture (outermost circle) (Fig. 4A). Local admixture plots showed a higher degree of shared ancestry between the Houghton and Wisconsin strains than between the Houghton and APU2 strains. Notably, a mosaic structure indicate introgression of large ancestral haploblocks among Houghton strains and the U.S. strains, supporting rare but consequential recombination driving population structure in *E. tenella*.

We employed incongruence in genetic distance to identify recombination points among genomes, using a line plot for the SNP alleles harbored by each strain (Fig. 4B). This identified substantial incongruence not only among Houghton and the U.S. strains but also within the U.S. strains. We also detected several cross-over points when comparing the Houghton and the U.S. strains. Strikingly, the APU2, Wisconsin, and Houghton strains differ completely between SNPs 45,924 and 49,751, resolving into only two major haplotypes. Elsewhere in the genome, the Wisconsin strain is much closer to APU2 (genetic distance between < 0.1 to ~ 0.5); thus, the three genomes have undergone a mosaic of introgression. Collectively, phylogenomic and recombination analysis suggested that although the U.S strains and Houghton strains have evolved independently, genetic admixture has shaped population genetic structure in this global parasite of poultry. In addition to the nuclear genome, apicomplexan parasites contain ~ 35 kb circular apicoplast genome, which is the remnant of a secondary endosymbiont^36^. Additionally, all Eimeria species harbor mitochondrial genomes and form concatemers of ~ 6200 bases^36^. These circular genomes are inherited maternally and do not undergo genetic recombination. Thus, we first checked the ancestral origin of Eimeria species by neighbor-joining analysis of the identified SNPs from organelle genomes using statistical distance and parsimony analyses (Fig. S5). By reference mapping with *E. tenella* Houghton and Eimeria species presented in Table S1, we identified 1185 and 598 high-confidence SNPs in apicoplast and mitochondrial genomes, respectively. Neighbor-joining analysis based on the identified SNPs from apicoplast, and mitochondrial genomes corresponded closely and showed that each Eimeria species were a descendant of a distinct matrilineage (Fig. S5). Two *E. tenella* isolates from the U.S. (ET_S13 and ERR296879) share the same allele of organelle genomes with Houghton strains despite being isolated from different geographical regions, indicating a single common maternal inheritance (Fig. S5). We utilized high-coverage Illumina reads from APU2 (ET_S13) to evaluate copy number of mitochondrial and apicoplast genomes between APU2 and Houghton strain. Of 307 million reads, the average read depth coverage for apicoplast sequences was 12,629 and 101,340 for mitochondrial sites; by contrast, the average read depth for nuclear genome coverage was 823 (Table 1). This suggests approximately 15 copies of the apicoplast, and 123 copies of the mitochondrial genomes occur for every copy of the nuclear genome of *E. tenella* APU2.

Genome variability can hinder drug development and can enable infectious organisms to escape immune defenses. Localized genomic variation may mediate host-parasite interactions. The adaptive immune response of vertebrates employs localized genomic diversity of functional genes and special antigen receptors to facilitate detection and efficient removal of foreign agents^37^. The SNPs and indels may engender functional differences. Thus, genetic variation may mediate immune evasion and drug resistance, but such variation has rarely been studied in complex and heterogeneous populations of parasites^38–40^. By identifying SNPs, our resource should help build high-density genetic maps of potentially informative genetic markers for a variety of applications^41,42^.

Recent studies from comparative genomics of closely related pathogens have revealed that genes in repeat-rich regions tend to evolve more rapidly than those in the rest of the genome^43^. SNPs can be employed to identify variations relevant for markers of drug response and other phenotypes, heralding major medical benefits^44^. It has been reported that random pairs of human genomes typically differ by approximately 0.1%^44^. Here, we determined almost an order of magnitude difference (0.088%) between two strains of *E. tenella,* and found most variation restricted to just a few, localized, telomeric regions. Occasional but consequential recombination may facilitate this pattern^45^.

Parasites infecting a given host likely generally share a close relationship, restricting the genetic consequence of sexual recombination owing to high levels of “selfing.” Mostly, biparental populations represent a very limited sample of genetic variation and have a high probability to carry the same alleles, whether measured at the scale of the locus or even the entire genome^46,47^. It has been also reported that *Eimeria* genome variability is due to short parasite generation times provide opportunities for rapid evolutionary events, i.e. development of differing levels of fecundity or pathogenicity^32^.

## Materials and methods

### *Eimeria tenella* oocyst isolation and DNA extraction

*Eimeria tenella* APU2 oocysts were recovered from a local broiler farm, isolated by limiting dilution, and maintained at our APDL laboratory, USDA ARS Beltsville by passage every 3–4 months in susceptible chickens. The oocysts were sporulated using standard procedures and stored in 2% K_2_CrO_4_ at 4 °C. *Eimeria tenella* APU2 oocysts (2.5 × 10^7^) were pelleted by centrifugation at 3000 rpm (1711 RCF or g force) for 10 min in a refrigerated centrifuge followed by treatment with 6.5% sodium hypochlorite for 30 min. to remove contaminating bacteria. The oocysts were washed 4 times by suspension in dH_2_O and centrifugation at 2000*g* for 10 min./wash. Pelleted oocysts were resuspended in Saline A (140 mM NaCl, 5 mM KCl, 4 mM NaHCO_3_, 1% dextrose, pH 7.0) and transferred to a glass mortar (Wheaton Instruments, Millville, NJ) for repeated grinding 50 times using a Wheaton Overhead Stirrer and a Teflon pestle (Wheaton). Released sporocysts were suspended in saline A and centrifuged at 3000 rpm for 10 min. The pellet was resuspended in 500 μl Inhibit-EX Buffer (Qiagen, Germantown, MD), transferred to a bead-beater tube with 200 mg 0.5 mm glass beads, and disrupted for 2 min. on a Mini Bead-Beater (Bio-Spec Products, Inc. Bartlesville, OK). The suspension was treated with 15 μl of proteinase K and 500 μl Buffer AL (Qiagen, Germantown, MD), followed by Phenol–Chloroform then Chloroform extraction. *E. tenella* DNA was ethanol precipitated; after centrifugation, the DNA pellet was washed in 70% ethanol, dried, and resuspended in 10 mM Tris pH 8.0. The integrity of DNA was analyzed with Genomic DNA ScreenTape on TapeStation (Agilent Technologies, Santa Clara, CA) showing a DNA Integrity Number of 7.1 with peak size of 12.9 kb.

### ONT and Illumina (Next-Seq) sequencing

An Oxford Nanopore sequencing library was prepared starting with 1.3 µg of genomic DNA using a ONT ligation sequencing kit SQK-LSK110. Approximately 250 ng of the total yield were run for 48 h on the MinION flow cell (R9.4.1) as per ONT sequencing protocol, that used for other similar genomes sequencing projects^48,49^. The QC and computational process of translating raw data to nucleotide sequence is of critical importance to the sequencing platforms produced by Oxford Nanopore Technologies (ONT)^50^. For Next-Seq sequencing, the library was created, starting with 100 ng of DNA (DIN 7.1), with an Illumina DNA Prep kit (Illumina, USA) in conjunction with dual-indexed paired end Illumina Indexes. Sequencing was performed using a total pooled loading concentration of 750 pM, with a 2% PhiX V3 spike-in, using 2 × 150 cycles (300 cycle) using P3 flow cell on Next-Seq 2000 sequencing system.

### Quality assessment and assembly 

The fast5 files were processed to gather sequences conversion as fastq with ONT guppy base-caller (version 6.5.7)^50^. These reads were subjected to processes with MaSuRCA assembler that combining Illumina reads and long reads from ONT and transforms large numbers of paired end reads into a much smaller number of longer ‘super-reads’. We used R9.4.1 flow cells combined with guppy base-caller with a high accuracy model providing reads with a modal accuracy of 97.6%, equivalent to a Phred score of Q16^51^. The error rate was further reduced by high long-read coverage and finally by polishing the assembly with Illumina high quality reads. Greater than 100-fold coverage using Oxford Nanopore technology helped ensure accuracy of the genome assembly.

### Chromosomal scaffolding of *E. tenella* APU2

The sequenced genome ordered with the help of available reference *E. tenella* Houghton^6^. We used RagTag toolset for automated assembly scaffolding using Minimap2, Unimap and Nucmer pipeline^13,52,53^. The sequenced *E. tenella* APU2 genome was oriented according to reference and ordered on chromosomes. This *E. tenella* APU2 chromosomes were analyzed for genome assembly consistency Jupiter plot, Circos to generate consistency between *E. tenella* Houghton and *E. tenella* APU2 genome assembly^54^. We compared our high-quality chromosomal-scale scaffolds to that previously reported for the Houghton strain for consistency and completeness.

### Structural variation detection

The GATK pipeline was used to identify genomic variants, including single nucleotide polymorphisms (SNPs) and insertions and deletions (Indels) using *E. tenella* APU2 with 307 million Illumina reads and *E. tenella* Houghton as reference genome^55,56^. The Picard toolkit utilities were used to perform related tasks such as processing and quality control of NGS data. The GATK “Variant Filtration” was applied to both SNPs and Indels output with "QD < 2.0", "FS > 60.0", "MQ < 40.0", "SOR > 4.0”, “MQRankSum < − 12.5", and "ReadPosRankSum < − 8.0" as suggested by GATK best practices pipeline. The analyzed results were further strengthened with quality filtrations with “PASS”, QC > 95 and “DP > 50” to remove any noise and having close to real SNPs. The processed quality filtered results of SNP-density were plotted SRplot on *E. tenella* Houghton 1 to 15 chromosomes. These SNP were subjected to annotate as per reference genomes coordinates predicted by AUGUSTUS pipeline and functionally annotation region with Snpdat^57^.

### Gene prediction, annotation and gene family analysis

The assembled chromosomal scaffolds were used on the trained prediction model using previously sequenced species *Eimeria tenella* Houghton as a model organism for AUGUSTUS gene prediction pipeline with RNA hints^10^. The genes were further annotated using Pfam database and GO-teams assignment^14,15^. We analyzed the predicted genes and classified them into different groups of gene family domain as predicted in Pfam that consisting of approximately 19,500 domains database. These Pfam were categorized based on genes and its family and selected high copy number of gene family mapped used for further analysis^58^ (Fig. S3).

### Population studies of *E. tenella* compared to sequenced genome

The availability of several strain sequences allowed us to evaluate various aspects of *E. tenella* genomics including population studies. The *E. tenella* Houghton genome was used as reference for this population studies^6^. We evaluated the available Illumina sequences of all the other genomes were obtained from Sequence Read Archive (https://www.ncbi.nlm.nih.gov/sra) and found the read generated was not enough. Therefore, lower the stringency (10 ×) of these population studies keeping other most of the parameter same as per read depth availability of *E. tenella* genomes. These short reads generated by Illumina paired-end reads (Table S1) were first mapped onto the *E. tenella* Houghton reference genome using the Burrows–Wheeler Aligner (BWA, v0.7.9)^59^ bwa-mem in default parameters and then converted to a bam file and sorted using SAMtools^60^. Sorted reads were then processed with Picard-1.8.4 (http://broadinstitute.github.io/picard) for soft-clipping and duplication. Local realignment around insertion/deletion and base quality score recalibration were performed using Genome Analysis Toolkit (GATK)^61^. GATK HaplotypeCaller was used to conduct the variant-calls with a read coverage ≥ 10×, a Phred scaled SNP quality of ≥ 30, and –ploidy = 1. Variants were converted into a table of bi-allelic SNPs using VCFtools^62^.

### Phylogenomic and network analysis

Genome-wide bi-allelic SNPs were converted into a FASTA file using a custom script and used for phylogenetic and network analysis using Molecular Evolutionary Genetic Analysis (MEGA) Version X^63^ and SplitsTree v.4.13.1^64^, respectively. Genome-wide SNPs from organelle genomes were directly incorporated into MEGAx for neighbor-joining analyses^63^ using both distance and parsimony methods after converted them into a fasta file and aligned with Clustal W/X^65^. One thousand bootstrap replicates were conducted, and consensus trees were drawn with an arbitrary root according to the bootstrap 50% majority rule. Neighbor-net method was used to construct an unrooted phylogenetic network with SNPs from the nuclear genome using the SplitsTree4 (v4.11.3) software program^64^ with 1000 bootstrap replicates.

### Homozygosity and heterozygosity calculation.

To calculate the proportions of heterozygous and homozygous SNPs present in each genome, SNP were filtered using SAMtools and BCFtools^66,67^ using the “mpileup” function and “ploidyfile” features and taking chromosomal ploidies into account. After calculating SNPs, heterozygous and homozygous SNPs were estimated in 5 kb blocks using custom Java scripts to generate histogram plots in Circos^54^. Red and blue colors indicate the presence of 90% or more heterozygous and homozygous SNPs, respectively, whereas yellow color was assigned otherwise.

### Ploidy determination

AGELESS software (http://ageless.sourceforge.net/) was used to calculate the ploidy of each specimen by dividing the chromosomes into 5 kb sliding windows and averaging the coverage within each window. The windows with zero coverage were not included in any further analyses due to sequencing noise or repeat regions^68^.

### Recombination crossovers point analysis.

Overall recombination pattern analysis was conducted using POPSICLE^34^ by aligning short-read sequences of *E. tenella* (*APU2*) genomes against the *E. tenella* Houghton reference in 5 kb sliding windows. For admixture analysis, we calculate the number of clusters K = 4 by determining the Dunn index after comparing the population structure with each cluster from K = 1 to 10. After assigning the optimal number of K, POPSICLE defines the admixture blocks by assigning each block to the clades using the current population genetic structure, followed by chromosomal painting in Circos plot^54^ with color assignment based on the number of K. The Recombination Analysis Tool (RAT)^69^ was used to calculate the genome-wide incongruence in the pairwise genetic distance to find out the cross-over points. We ran the RAT software with an average of 82% sequence identity and 92% jump to the next window with a sliding window of 5 kb. If the genetic distance in the current window is below the lower threshold parameter and either one of the next two windows is above the upper threshold parameter, then the current sequence is flagged as a possible recombinant^69^.

### Supplementary Information

Supplementary Information.

Supplementary Table S2.

Supplementary Table S3.

## Data Availability

The reported *E. tenella* APU2 chromosomal scale assembly is available at NCBI with Bio-project PRJNA929509 with the accession number CP118642-CP118656 for each of the 1 to 15 chromosomes and raw data with SRA numbers SSR24971025 and SSR24971026.

## References

[1] da Cunha AF, Santin E, Kogut M (2020). Editorial: Poultry coccidiosis: Strategies to understand and control. Front. Vet. Sci..

[2] Amarasinghe SL (2020). Opportunities and challenges in long-read sequencing data analysis. Genome Biol..

[3] Khan AR, Pervez MT, Babar ME, Naveed N, Shoaib M (2018). A comprehensive study of de novo genome assemblers: Current challenges and future prospective. Evol. Bioinform. Online.

[4] Blake DP (2012). EmaxDB: Availability of a first draft genome sequence for the apicomplexan *Eimeria*
*maxima*. Mol. Biochem. Parasitol..

[5] Reid AJ (2014). Genomic analysis of the causative agents of coccidiosis in domestic chickens. Genome Res..

[6] Aunin E (2021). The complete genome sequence of *Eimeria tenella* (Tyzzer 1929), a common gut parasite of chickens. Wellcome Open Res..

[7] Zappala Z, Montgomery SB (2016). Non-coding loss-of-function variation in human genomes. Hum. Hered..

[8] Koren S (2017). Canu: scalable and accurate long-read assembly via adaptive k-mer weighting and repeat separation. Genome Res..

[9] Nishimura O, Hara Y, Kuraku S (2017). gVolante for standardizing completeness assessment of genome and transcriptome assemblies. Bioinformatics.

[10] Stanke M, Schoffmann O, Morgenstern B, Waack S (2006). Gene prediction in eukaryotes with a generalized hidden Markov model that uses hints from external sources. BMC Bioinform..

[11] Zimin AV (2013). The MaSuRCA genome assembler. Bioinformatics.

[12] Simao FA, Waterhouse RM, Ioannidis P, Kriventseva EV, Zdobnov EM (2015). BUSCO: Assessing genome assembly and annotation completeness with single-copy orthologs. Bioinformatics.

[13] Alonge M (2022). Automated assembly scaffolding using RagTag elevates a new tomato system for high-throughput genome editing. Genome Biol..

[14] Sonnhammer EL, Eddy SR, Durbin R (1997). Pfam: A comprehensive database of protein domain families based on seed alignments. Proteins.

[15] Mistry J (2021). Pfam: The protein families database in 2021. Nucleic Acids Res..

[16] Chan PP, Lin BY, Mak AJ, Lowe TM (2021). tRNAscan-SE 2.0: Improved detection and functional classification of transfer RNA genes. Nucleic Acids Res..

[17] Stanke M (2006). AUGUSTUS: Ab initio prediction of alternative transcripts. Nucleic Acids Res..

[18] Yeoh LM, Lee VV, McFadden GI, Ralph SA (2019). Alternative splicing in apicomplexan parasites. mBio.

[19] Beziau A, Brand D, Piver E (2020). The role of phosphatidylinositol phosphate kinases during viral infection. Viruses.

[20] Mitchell A (2015). The InterPro protein families database: The classification resource after 15 years. Nucleic Acids Res..

[21] Harris MA (2004). The Gene Ontology (GO) database and informatics resource. Nucleic Acids Res..

[22] Emms DM, Kelly S (2019). OrthoFinder: Phylogenetic orthology inference for comparative genomics. Genome Biol..

[23] Heitlinger E, Spork S, Lucius R, Dieterich C (2014). The genome of *Eimeria*
*falciformis*—reduction and specialization in a single host apicomplexan parasite. BMC Genom..

[24] Prakashbabu BC (2017). Species occurrence varies between geographic regions and poultry production systems and may influence parasite genetic diversity. Vet. Parasitol..

[25] Blake DP (2015). Population, genetic, and antigenic diversity of the apicomplexan and their relevance to vaccine development. Proc. Natl. Acad. Sci. USA.

[26] Ramana J, Gupta D (2009). ProtVirDB: A database of protozoan virulent proteins. Bioinformatics.

[27] Takai D, Jones PA (2002). Comprehensive analysis of CpG islands in human chromosomes 21 and 22. Proc. Natl. Acad. Sci. USA.

[28] Miles A (2016). Indels, structural variation, and recombination drive genomic diversity in *Plasmodium*
*falciparum*. Genome Res..

[29] Girgis HZ (2015). Red: An intelligent, rapid, accurate tool for detecting repeats de-novo on the genomic scale. BMC Bioinform..

[30] Avvaru AK, Sowpati DT, Mishra RK (2018). PERF: An exhaustive algorithm for ultra-fast and efficient identification of microsatellites from large DNA sequences. Bioinformatics.

[31] Klotz C, Gehre F, Lucius R, Pogonka T (2007). Identification of *Eimeria*
*tenella* genes encoding for secretory proteins and evaluation of candidates by DNA immunisation studies in chickens. Vaccine.

[32] Blake DP, Worthing K, Jenkins MC (2020). Exploring *Eimeria* genomes to understand population biology: Recent progress and future opportunities. Genes.

[33] Blake DP (2015). Population, genetic, and antigenic diversity of the apicomplexan *Eimeria*
*tenella* and their relevance to vaccine development. Proc. Natl. Acad. Sci. USA.

[34] Shaik JS, Khan A, Grigg ME (2018). POPSICLE: A software suite to study population structure and ancestral determinates of phenotypes using whole genome sequencing data. bioRxiv.

[35] Dunn JC (1973). A fuzzy relative of the ISODATA process and its use in detecting compact well-separated clusters. J. Cybern..

[36] Ogedengbe ME, El-Sherry S, Whale J, Barta JR (2014). Complete mitochondrial genome sequences from five *Eimeria* species (Apicomplexa; Coccidia; Eimeriidae) infecting domestic turkeys. Parasit. Vectors.

[37] Hannigan GD (2017). Evolutionary and functional implications of hypervariable loci within the skin virome. PeerJ.

[38] Minot S, Grunberg S, Wu GD, Lewis JD, Bushman FD (2012). Hypervariable loci in the human gut virome. Proc. Natl. Acad. Sci. USA.

[39] Wood CL (2007). Parasites alter community structure. Proc. Natl. Acad. Sci. USA.

[40] Zilversmit MM (2013). Hypervariable antigen genes in malaria have ancient roots. BMC Evol. Biol..

[41] Cheeseman K, Weitzman JB (2015). Host-parasite interactions: An intimate epigenetic relationship. Cell. Microbiol..

[42] Hong YH, Kim ES, Lillehoj HS, Lillehoj EP, Song KD (2009). Association of resistance to avian coccidiosis with single nucleotide polymorphisms in the zyxin gene. Poult. Sci..

[43] Huang X (2016). Identification of highly variable supernumerary chromosome segments in an asexual pathogen. PLoS One.

[44] Shastry BS (2007). SNPs in disease gene mapping, medicinal drug development and evolution. J. Hum. Genet..

[45] Allen PC, Fetterer RH (2002). Recent advances in biology and immunobiology of *Eimeria* species and in diagnosis and control of infection with these coccidian parasites of poultry. Clin. Microbiol. Rev..

[46] Hurst GD, Frost CL (2015). Reproductive parasitism: Maternally inherited symbionts in a biparental world. Cold Spring Harb. Perspect. Biol..

[47] Huang X (2011). Analysis of natural allelic variation in *Arabidopsis* using a multiparent recombinant inbred line population. Proc. Natl. Acad. Sci. USA.

[48] Srivastava SK (2020). Draft genome resource for the ex-types of *Phytophthora*
*ramorum*, *P.*
*kernoviae*, and *P*. *melonis*, species of regulatory concern, using ultra-long read MinION nanopore sequencing. Mol. Plant Microbe Interact..

[49] Srivastava SK, Zeller KA, Sobieraj JH, Nakhla MK (2021). Genome resources of four distinct pathogenic races within *Fusarium*
*oxysporum* f. sp. *vasinfectum* that cause vascular wilt disease of cotton. Phytopathology.

[50] Wick RR, Judd LM, Holt KE (2019). Performance of neural network basecalling tools for Oxford Nanopore sequencing. Genome Biol..

[51] Ni Y, Liu X, Simeneh ZM, Yang M, Li R (2023). Benchmarking of Nanopore R10.4 and R9.4.1 flow cells in single-cell whole-genome amplification and whole-genome shotgun sequencing. Comput. Struct. Biotechnol. J..

[52] Li H (2018). Minimap2: Pairwise alignment for nucleotide sequences. Bioinformatics.

[53] Marcais G (2018). MUMmer4: A fast and versatile genome alignment system. PLoS Comput. Biol..

[54] Krzywinski M (2009). Circos: An information aesthetic for comparative genomics. Genome Res..

[55] Van der Auwera GA (2013). From FastQ data to high confidence variant calls: The Genome Analysis Toolkit best practices pipeline. Curr. Protoc. Bioinform..

[56] DePristo MA (2011). A framework for variation discovery and genotyping using next-generation DNA sequencing data. Nat. Genet..

[57] Doran AG, Creevey CJ (2013). Snpdat: Easy and rapid annotation of results from de novo snp discovery projects for model and non-model organisms. BMC Bioinform..

[58] Srivastava SK (2014). The genome sequence of the fungal pathogen *Fusarium*
*virguliforme* that causes sudden death syndrome in soybean. PLoS One.

[59] Li H, Durbin R (2009). Fast and accurate short read alignment with Burrows–Wheeler transform. Bioinformatics.

[60] Li H (2011). A statistical framework for SNP calling, mutation discovery, association mapping and population genetical parameter estimation from sequencing data. Bioinformatics.

[61] McKenna A (2010). The Genome Analysis Toolkit: A MapReduce framework for analyzing next-generation DNA sequencing data. Genome Res..

[62] Danecek P (2011). The variant call format and VCFtools. Bioinformatics.

[63] Kumar S, Stecher G, Li M, Knyaz C, Tamura K (2018). MEGA X: Molecular evolutionary genetics analysis across computing platforms. Mol. Biol. Evol..

[64] Huson DH, Bryant D (2006). Application of phylogenetic networks in evolutionary studies. Mol. Biol. Evol..

[65] Larkin MA (2007). Clustal W and Clustal X version 2.0. Bioinformatics.

[66] Li H (2009). The sequence alignment/map format and SAMtools. Bioinformatics.

[67] Narasimhan V (2016). BCFtools/RoH: A hidden Markov model approach for detecting autozygosity from next-generation sequencing data. Bioinformatics.

[68] Inbar E (2019). Whole genome sequencing of experimental hybrids supports meiosis-like sexual recombination in *Leishmania*. PLoS Genet..

[69] Etherington GJ, Dicks J, Roberts IN (2005). Recombination Analysis Tool (RAT): A program for the high-throughput detection of recombination. Bioinformatics.

