# Supplementary material for: Chromosomal scale assembly reveals localized structural variants in avian caecal coccidian parasite Eimeria tenella
Source: Sci Rep. 2023 Dec 20;13:22802. doi: 10.1038/s41598-023-50117-0 (PMC10739835; doi:10.1038/s41598-023-50117-0)

**Additional information.**

**Table S1.** Short-read sequences used in *E. tenella* strains population study.

**Supportive data Table Legends.**

**Table S2**. Genome wide annotations of *E. tenella* APU2 genes-based SNPs with Pfam and GO annotations.xlsx.

**Table S3.** *E. tenella* Houghton and *E. tenella* APU2 Highly variable top 30 Pfam gene family with number of SNPs.xlsx

**Supportive Figure Legends.**

**Figure S1.** Workflow for the *E. tenella* APU2 genome sequencing, assembly, and downstream analyses.

**Figure S2. A).** BUSCO assessment for *E. tenella* APU2 sequenced genome. **B).**  Pfam annotations with number 5 and above annotation comparison between two reference chromosomal level assembly of *E. tenella* strains.

**Figure S3** Overview of the functional annotations Pfam (≥15 genes) and GO-teams (≥5 genes) in *E. tenella* APU2 classification based on Pfam annotation and GO annotation.

**Figure S4** Chromosomal distribution of CpG sites between *E. tenella* Houghton and *E. tenella* APU2 genome.

**Figure S5.** Phylogenomic analysis of organelle genomes of *Eimeria* species showed the monophyletic clustering of all *E. tenella* sequences analyzed in this study.

**Figure S6** Phylogeny analysis of important avian parasites *E. mitis*, *E. brunetti*, *E. praecox*, *E. maxima*, *E. acervulina*, *E. tenella*-APU2, *E. necatrix*, *E. falciformis* and *T. gondii.*

**Table S1.** Short-read sequences used in *E. tenella* strains population study.

| **SRA*** | **strain** | **sample name** | ***Eimeria*** | **center name** |
| --- | --- | --- | --- | --- |
|  | Maryland, USA | ET_S13** (APU2) | *E. tenella* | Animal Parasitic Disease Laboratory, ARS, USDA |
| ERR019311 | Houghton | ERS004356 | *E. tenella* | Wellcome Sanger Institute |
| ERR019312 | Houghton | ERS004356 | *E. tenella* | Wellcome Sanger Institute |
| ERR296541 | Nippon | Nippon Nt2 | *E. tenella* | Royal Veterinary College |
| ERR019305 | Houghton | ERS006630 | *E. tenella* | Wellcome Sanger Institute |
| ERR296879 | Wisconsin, USA |  | *E. tenella* | Royal Veterinary College |
| ERR019310 | Houghton | ERS002383 | *E. tenella* | Wellcome Sanger Institute |
| ERR019307 | Houghton | ERS006630 | *E. tenella* | Wellcome Sanger Institute |
| ERR019306 | Houghton | ERS006630 | *E. tenella* | Wellcome Sanger Institute |
| ERR019309 | Houghton | ERS002383 | *E. tenella* | Wellcome Sanger Institute |
| ERR6054480 | Houghton | Cumbria, Hawkshead | *E. tenella* | Royal Veterinary College |
| ERR6054482 | Houghton | Cumbria, Hawkshead | *E. tenella* | Royal Veterinary College |
| ERR6054483 | Houghton | Cumbria, Hawkshead | *E. tenella* | Royal Veterinary College |
| ERR6054481 | Houghton | Cumbria, Hawkshead | *E. tenella* | Royal Veterinary College |
| SRR23018153 | Houghton strain | China, Nanning | *E. tenella* | Guangxi University |
| SRR23018155 | Houghton strain | China, Naning | *E. tenella* | Guangxi University |
| ERR357127 | Houghton |  | *E. acervulina* | King Abdullah University of Science and Technology |
| ERR357128 | Weybridge |  | *E. maxima* | King Abdullah University of Science and Technology |
| ERR357129 | Houghton |  | *E. necatrix* | King Abdullah University of Science and Technology |
| ERR357130 | Houghton |  | *E. brunetti* | King Abdullah University of Science and Technology |
| ERR357191 | Houghton |  | *E. mitis* | King Abdullah University of Science and Technology |
| ERR357192 | Houghton |  | *E. praecox* | King Abdullah University of Science and Technology |
| ERR4552264 |  | OTUx | *Eimeria sp.* OTU-X | Royal Veterinary College |
| ERR4552265 |  | OTUy | *Eimeria sp. OTU-Y* | Royal Veterinary College |
| ERR4552266 |  | OTUz | *Eimeria sp.* OTU-Z | Royal Veterinary College |

* Short-read sequences were downloaded from the Sequence Read Archive (SRA, https://www.ncbi.nlm.nih.gov/sra).** The *E. tenella*  APU2 sequencing specimens were acquired from a local poultry form and maintained at APDL, USDA ARS Beltsville

**Supportive Figures**

**Figure S1.** Workflow for the *E. tenella* APU2 genome sequencing, assembly, and downstream analyses. The Sequencing Step involved sequencing of *E. tenella* APU2 with two different Technology, Illumina NextSeq (2^nd^ Generation) and Oxford Nanopore MinION (3^rd^ Generation). Followed by downstream analyses.

**
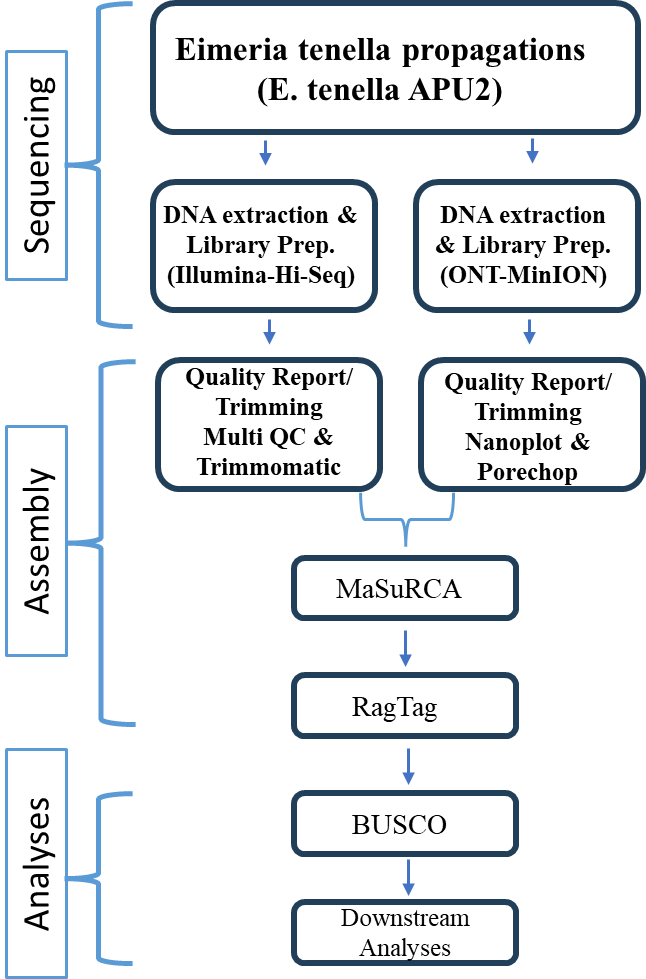
**

**Figure S2. A).** BUSCO assessment for *E. tenella* APU2 sequenced genome. **B).**  Pfam annotations with number 5 and above annotation comparison between two reference chromosomal level assembly of *E. tenella* strains.

**A).** BUSCO assessment for *E. tenella* APU2 sequenced genome.


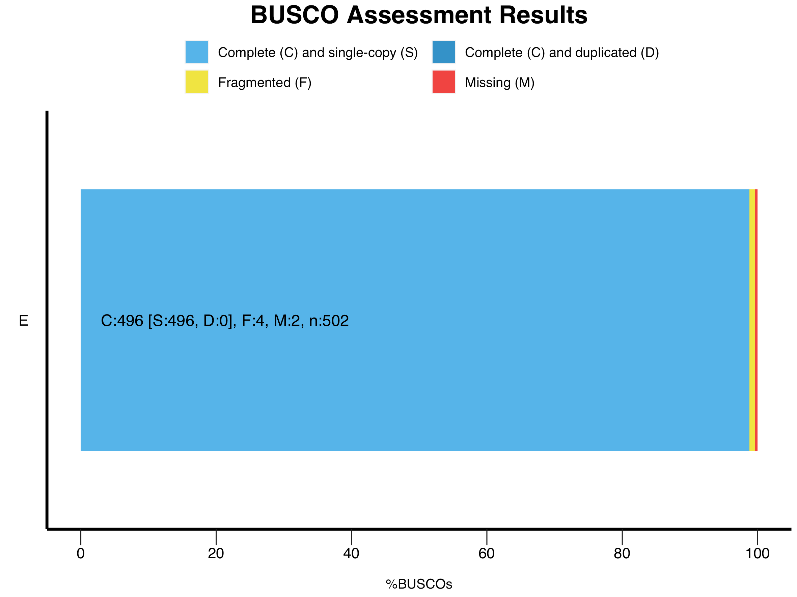


**B).**  Pfam annotation comparison between two reference chromosomal level assembly of *E. tenella* strains

**Figure S3** Overview of the functional annotations Pfam (≥15 genes) and GO-teams (≥5 genes) in *E. tenella* APU2. These proteins were classified based on Pfam annotation and GO annotation assignment to gain an overview of their biological function classification genome-wide and across the chromosomes. The GO-teams were generated based on InterPro2GO mapping. Highly variable 30 Pfam annotated variable genes between *E tenella* Houghton and *E. tenella* APU2 strains.

**Figure S4** Chromosomal distribution of CpG sites between *E. tenella* Houghton and *E. tenella* APU2 genome. The Observed number of CpG patterns in a window is the number of times a 'C' is found followed immediately by a 'G' represented as per chromosomes a crossed the *E. tenella* strain.

**
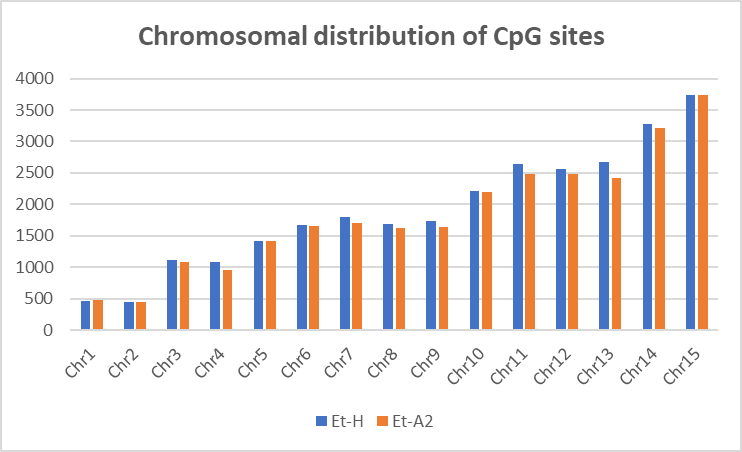
**

**Figure S5.** Phylogenomic analysis of organelle genomes of *Eimeria* species showed the monophyletic clustering of all *E. tenella* sequences analyzed in this study. Complete apicoplast **(A)** and mitochondria **(B)** genome phylogenies of *Eimeria* were constructed by downloading available whole genome sequences in Sequence Read Archive (https://www.ncbi.nlm.nih.gov/sra) and by comparing with the current chromosomal scaffolding of *E. tenella*  strain APU2 (ET_S13). Apicoplast and mitochondrial reference genomes were obtained from the Assembly accession number: GCA_905310635.1(4). Single nucleotide polymorphisms were identified by reference mapping and variant calling using the GATK pipeline(46). The phylogram was obtained by an unrooted neighbor-joining tree using Mega with a 50% majority rule and 1000 bootstrap replicates (indicated at each node as percentage values). *Eimeria* species are placed in brackets.


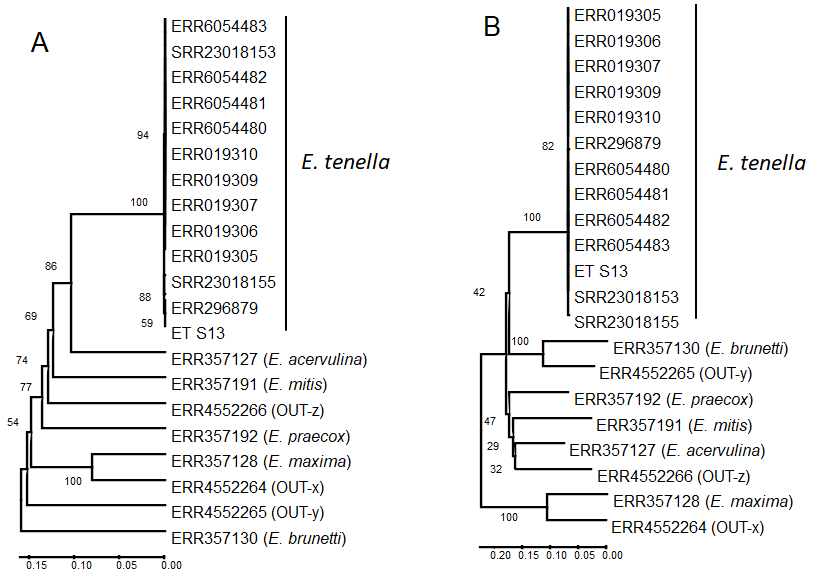


**Figure S6** Phylogeny analysis of avian parasites *E. tenella* APU2, *T. gondii*, *E. acervuline*, *E. brunetti*, *E. maxima*, *E. mitis*, *E. nectrix*, *E. praecox*, and *E. falciformis* shows E. necatrix are in one clade share close relatioship with *E. tenella* APU2, and *E. tenella* Houghton, likewise *E. maxima* and *E. acervulina* are in anther close group.


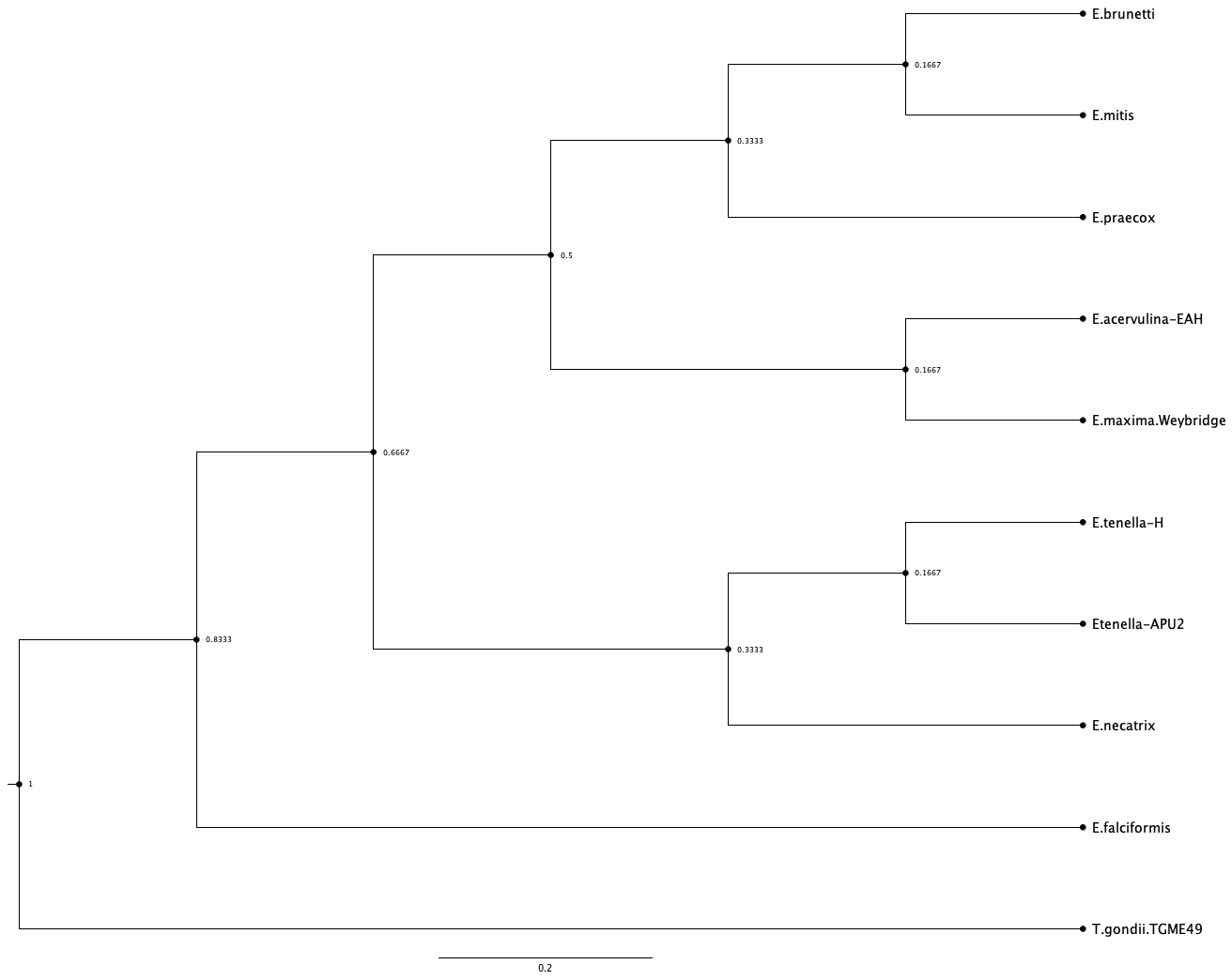

Supplement: Supplementary file 1 — Supplementary Information. [file 41598_2023_50117_MOESM1_ESM.docx]
